# High pH Stress Affects Root Morphology and Nutritional Status of Hydroponically Grown Rhododendron (*Rhododendron* spp.)

**DOI:** 10.3390/plants9081019

**Published:** 2020-08-12

**Authors:** Ashley J. Turner, Camila I. Arzola, Gerardo H. Nunez

**Affiliations:** Horticultural Sciences Department, University of Florida, Gainesville, FL 32611, USA; ashleyturner@ufl.edu (A.J.T.); carzola@ufl.edu (C.I.A.)

**Keywords:** ericaceae, calcifuge, cluster roots

## Abstract

Rhododendrons (*Rhododendron* spp.) are ornamental plants in the family Ericaceae that thrive in acidic soils and are challenged by neutral or alkaline soils. This soil requirement limits the locations where rhododendrons can be grown and causes chlorosis, diminished growth, and low survival when rhododendrons are grown in high pH soils. While growth and survival impacts are widely documented, little is known about how high pH soils cause these symptoms in rhododendrons. We hypothesized that high pH stress impacts root form and function, leading to nutrient deficiencies that limit plant growth. We tested this hypothesis in a hydroponic experiment. “Mardi Gras” rhododendron liners were grown in a complete nutrient solution at pH 5.5 (optimum pH) or pH 6.5 (high pH) for 49 days. Biomass accumulation, nutrient uptake and concentration, and root stress were assessed. High pH nutrient solutions diminished leaf and root growth. Plants grown in high pH nutrient solutions developed clusters of short, highly branched roots. Plants grown in optimum pH did not exhibit this morphology. High pH affected the uptake and translocation of most essential nutrients. S and Mn deficiencies likely limited plant growth. High pH had a nuanced effect on root oxidative status. These results suggest that rhododendron root morphology and nutrient uptake are directly affected by high pH and that aboveground symptoms might be a consequence of impaired root function.

## 1. Introduction

Rhododendrons (*Rhododendron* spp.) are ornamental plants in the family Ericaceae. While some taxa exhibit wide pH tolerance [1], most rhododendrons thrive in acidic soils (pH 5.0 to pH 6.0) and are challenged by neutral or alkaline soils [2]. Acidic soils are uncommon in gardens and landscapes, which limits the locations where rhododendrons can be planted [3].

Cultivation in high pH soils elicits several responses in rhododendron and other calcifuge plants. The most common stress symptom is mild to severe chlorosis. Chlorosis has been previously used as a tool to assess plant stress and select genotypes with high pH tolerance [3,4,5]. While chlorosis has been commonly ascribed to Fe deficiency, Fe concentration and Fe content were not measured in these studies. Additionally, research with other calcifuge plants suggests other nutrients might be involved. In blueberry (*Vaccinium* spp. section *cyanococcus*), chlorosis has been observed in plants grown in high pH soils, even though leaf Fe concentrations were not affected [6,7]. In cranberry (*Vaccinium macrocarpon*), shoot Fe, Mn, and Zn were lower in response to high pH nutrient solutions [8]. Further research is necessary to identify the cause and relevance of rhododendron chlorosis in high pH soils.

Another common symptom of high pH stress is diminished growth. Rhododendrons grow less [2,3] and have higher mortality rates [5,9] in high pH soils than in acidic soils. Similar responses have been observed in blueberry, cranberry [8], heath (*Erica* spp.), and heather (*Calluna vulgaris*) [10].

Weak, chlorotic growth diminishes the value of ornamental and edible ericaceous plants. Research with other calcifuge plants suggests that root system morphology and physiology might be the link between nutritional deficiencies, impaired growth, and low survival. High pH soils reduce root growth in blueberry [6,8] and cranberry [8]. Smaller root systems have limited access to water and nutrients in the soil [11], leading to other downstream symptoms. Size-independent responses have also been observed. Blueberry roots upregulate Fe uptake [12] and cranberry roots reduce their organic acid concentrations [13] and increase branching [14] in high pH soils or nutrient solutions. It is unknown whether size-dependent or -independent root responses are involved in the rhododendron response to high pH.

This research aims to describe how high pH nutrient solutions affect rhododendrons. We hypothesized that high pH stress impacts root form and function, leading to nutrient deficiencies that limit plant growth. We tested this hypothesis in a hydroponic experiment.

## 2. Results

### 2.1. Growth, Dry Weight, and Chlorosis

**Figure 1:**
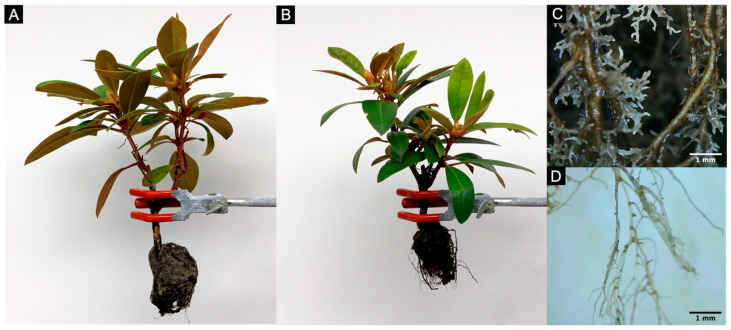
“Mardi Grass” rhododendron (*Rhododendron* spp.) after 49 days of growth in nutrient solution at pH 5.5 (**A**) or pH 6.5 (**B**). Plants developed clusters of short, highly-branched first and second order roots at pH 6.5 (**C**), but not at pH 5.5 (**D**).

“Mardi Gras” rhododendron liners were acclimated to a hydroponic growth system for 22 days. At the end of acclimation, plants weighed 38.14 ± 2.53 g (mean ± standard error). Subsequently, plants were transferred to optimum (pH 5.5) or high pH (pH 6.5) nutrient solutions. All plants grew during the 49-day treatment period, but plants in pH 5.5 exhibited higher relative growth rates than plants in pH 6.5 (0.40 vs. 0.17, *p* = 0.024). At the end of the experiment, plants grown in optimum pH solutions were larger than plants grown in high pH solutions (Table 1). Roots and leaves were the most affected organs. Stems were not affected, and plants did not bloom during the experiment. Plants grown in pH 5.5 had larger root systems than plants grown in pH 6.5, but root to shoot ratios were not different between the treatments. Plants grown in optimal pH solutions did not exhibit root morphological changes during the experiment. In contrast, plants grown in high pH solutions developed short, highly-branched first and second order roots clustered together in the basal areas of higher-order roots (Figure 1). Plants grown in pH 5.5 had larger total leaf area and leaf dry weight than plants grown in pH 6.5. Chlorosis was not detected visually or through transmittance (SPAD average = 27.21, *p* = 0.950) or image analysis-based methods (Dark Green Color Index average = 0.48, *p* = 0.950) in either treatment.

### 2.2. Nutrient Uptake and Content

**Figure 2:**
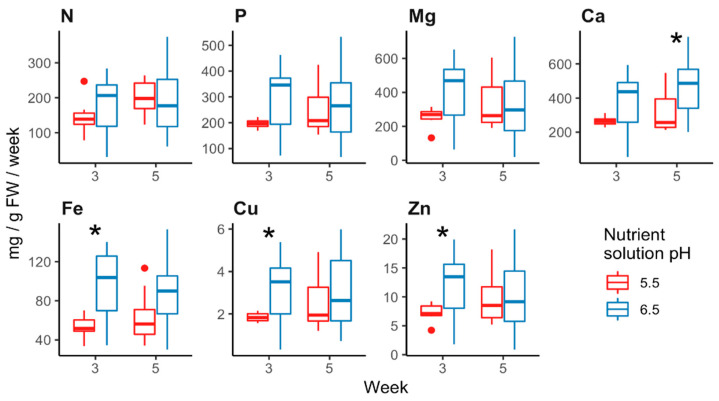
Weekly nutrient uptake by “Mardi Gras” rhododendron grown in nutrient solution at pH 5.5 or pH 6.5. Element depletion from the nutrient solution was divided by plant fresh weight to account for size differences. Asterisks indicate significant differences at α = 0.05. Dots indicate outliers.

Weekly nutrient uptake was measured twice (during weeks 3 and 5 of the treatment period). To account for size differences, uptake was divided by whole plant fresh weight. N, P, and Mg uptake were not affected by nutrient solution pH (Figure 2). Plants took up more Ca (week 5), Fe, Cu, and Zn (week 3) when grown in high pH nutrient solutions than when grown in optimum pH solutions. Nutrient solution pH did not affect ferric chelate reductase activity 16 and 47 days after the start of treatments (*p* > 0.136 in all cases). Nitrate reductase activity was not affected by nutrient solution pH on day 28 (*p* = 0.087). Acid phosphatase activity was not affected by nutrient solution pH on day 22 (*p* = 0.663). Both of these enzymes exhibited higher activity in plants grown at pH 6.5 than in plants grown at pH 5.5 on day 48 (*p* < 0.049 in all cases). Enzymatic activity averages can be found in Table S1.

Nutrient solution pH affected macro and micronutrient concentrations in roots and leaves (Table 2). Roots of plants grown in high pH solutions had lower N, S, Cu, and Zn concentrations and higher Fe and B concentrations than roots of plants grown in optimum pH solutions. Leaves of plants grown in high pH solutions had lower P, Mg, S, Mn, and B concentrations and higher N concentrations than leaves of plants grown in optimum pH solutions. High pH solutions had the same effect in S concentrations in leaves and roots but contrasting effects in all other nutrient concentrations. Leaf and root Ca concentrations were not affected.

Nutrient solution pH also affected organ nutrient content (Table 3). Roots of plants grown in high pH solutions contained less N, S, Cu, Mn, and Zn than roots of plants grown in optimum pH solutions. Leaves of plants grown in high pH solutions contained less Mg, S, and Mn than leaves of plants grown in optimum pH solutions. Leaf and root P, Ca, Fe, and B contents were not affected by nutrient solution pH.

### 2.3. Root Stress

Nutrient solution pH did not affect root membrane integrity or oxidative status consistently, as effects observed in one sampling date were absent in another date (Figure S1). Roots of plants grown in high pH solutions exhibited more electrolyte leakage than roots of plants grown in optimum pH solutions 21 days after the start of the experiment (20.97% vs. 33.55%, *p* = 0.002). Roots of plants grown in high pH solutions exhibited higher catalase activity than roots of plants grown in optimum pH solutions 19 days after the start of the experiment (0.13 mmol H_2_O_2_ mg^−1^ fresh weight (FW) min^−1^ vs. 0.23 H_2_O_2_ mg^−1^ FW min^−1^, *p* = 0.039). Electrolyte leakage on day 49 and catalase activity on day 4 were not significantly affected. Lipid peroxidation and proline concentration were not affected by nutrient solution pH in any of the dates tested.

## 3. Discussion

While the negative effects of growth in high pH soils are widely documented in rhododendron and other calcifuge plants, very little is known about the mechanism that causes these symptoms. Previously, rhododendron responses to high pH have been studied using CaCO_3_ amendments in soils or substrates [2,15], but carbonates elicit both pH-dependent and pH-independent responses [16,17,18]. Thus, in the present experiment, we used hydroponic solutions where pH can be adjusted without carbonates to study the rhododendron response to high pH.

Rhododendron in high pH nutrient solutions grew less than rhododendron in optimum pH solutions in this experiment. These results are consistent with previous findings in rhododendron [2,3] and other calcifuge plants [8,10,14], and they imply the existence of one or more factors that limit growth. Our results suggest that root morphology and function might be the limiting factors. Rhododendrons grown in high pH nutrient solutions exhibited smaller root systems than plants grown in optimum pH at the end of the experiment. Even though root to shoot ratios were not affected by nutrient solution pH here and elsewhere [19], small root systems appear central to the high pH stress response because (1) high pH soils or nutrient solutions have direct contact with roots, and (2) root symptoms were more drastic than symptoms in other organs. Previous research has shown that small root systems have limited ability to access [11] or take up [20] water and nutrients in the soil. Therefore, small root systems could be a reason for the nutrient deficiencies and diminished shoot growth observed in this experiment.

High pH stress—or nutritional deficiencies caused by high pH stress—also led to the development of abnormal root system morphology. While plants grown in optimum pH nutrient solutions exhibited canonical rhododendron root morphology [21], plants grown in high pH solutions developed clusters of short, highly branched roots that resemble stress-adaptive root morphology in other plant species (e.g., proteoid roots in the family Proteaceae, cluster roots in the family Casuarinaceae, Mimosaceae, Fabaceae, Myricaceae, and Moraceae) [22]. High pH nutrient solutions increased root branching in cranberry, but it is unclear if the morphology documented by Finn et al. [14] matches our observations. Unfortunately, the imaging methods used in this experiment were not suitable for quantitative analysis of root morphology. To the best of our knowledge, this is the first report of root clusters (following the nomenclature of Lamont [20]) in the family Ericaceae. Proteoid and cluster roots have high surface area-to-mass ratios which enhance their exudation, nutrient solubilization, and nutrient uptake [23]. For example, proteoid roots are capable of acidifying the rhizosphere through H^+^ [24] or organic acid [25] extrusion. Similar acidification capacity would enable rhododendrons to create a more favorable rhizosphere microenvironment by avoiding high pH stress. Rhizosphere acidification has been previously observed in rhododendron [3] and other calcifuge plants [6,26], but root morphology was not studied simultaneously. Further research should study this abnormal root morphology through quantitative methods and explore the connection between rhizosphere acidification and root clusters in rhododendron.

Another possibility is that root clusters might be a nutrient deficiency response. Other species develop proteoid or cluster roots when plant growth is limited by N, P, or Fe [23]. Leaf N and Fe concentrations were not affected by nutrient solution pH in this experiment. On the other hand, plants grown in high pH solutions exhibited lower leaf P concentrations than plants grown in optimum pH solutions. P limitations have been previously documented in blueberry [7] and other calcifuge species [10] grown in high pH soils. Hence, root cluster development could be related to P deficiency. At root level, P content and P concentration were not different between plants grown at high pH and plants grown at optimum pH, suggesting root clusters might play a role in P uptake. Other responses to P deficiency, such as high acid phosphatase activity and leaf reddening were not observed. Since root sampling for acid phosphatase activity assays did not discriminate between root types (cluster vs. non-cluster), these measurements are not informative about cluster root function in this experiment. Considering differences between proteoid and non-proteoid [22] and cluster and non-cluster roots [27] have been extensively documented in other species, further research with rhododendron roots is warranted.

Other nutritional deficiencies were also observed. Leaf S and Mn concentrations and contents were lower in plants grown in high pH nutrient solutions than in plants grown in optimum pH solutions. Root S and Mn content were similarly affected. Blueberry and cranberry also exhibited lower shoot Mn concentrations in high pH soils solutions than in low pH ones [8]. Considering that biomass accumulation, nutrient concentration, and nutrient content were negatively impacted, it is possible that S and/or Mn deficiency were limiting to growth [28]. Other results were less intuitive and must be considered in the context of the different growth rates in each treatment. Some elements were deficient in leaves but not in roots (Mg), others deficient in roots but not in leaves (N, Cu, Zn), and others exhibited differences in nutrient concentration but not in nutrient content (B). Since plants grown in optimum pH nutrient solutions were larger than plants grown in high pH nutrient solutions at the end of the experiment, leaf nutrient concentrations in the former group could have suffered a “dilution effect”. This effect could explain leaf concentration trends, particularly for N which is mobile within the plant [29]. These results underscore the complex, widespread impact high pH has in nutrient uptake and translocation by rhododendron.

In this experiment, plants grown in high pH nutrient solutions were not Fe deficient and they did not develop chlorosis. Fe deficiency is commonly observed in plants grown in high pH soils [8], because Fe is less bioavailable under these conditions [30]. Chlorosis is an Fe deficiency symptom that has been used to identify rhododendrons that can tolerate high pH soils [3,4,5,15]. It is possible that deficiency was avoided because stressed plants grew very slowly, Fe was delivered as a pH-stable chelate in the nutrient solution, or the treatment period was short. Nevertheless, our results suggest that chlorosis might not be an informative phenotype to select rhododendrons that tolerate high pH, because high pH stress caused numerous stress responses in absence of this leaf symptom.

Nutrient solution pH might have also affected nutrient bioavailability and translocation. Root Fe concentrations were higher in rhododendrons grown in high pH nutrient solutions than in rhododendrons grown in low pH solutions, but leaf concentration was not affected. This suggests that Fe in the roots was either not bioavailable or that translocation was impaired. While high Fe concentrations in roots could be caused by higher Fe uptake at pH 6.5, unaffected ferric chelate reductase activity rates suggest some of this Fe was likely unavailable to the plant. Rhododendron and several other plants have total and “active” Fe pools [15,31]. The former includes all the Fe present in an organ, while the latter includes only the bioavailable fraction. Fe precipitation is strongly pH dependent [30]. Thus, high pH nutrient solutions could have caused apoplastic Fe precipitation, making Fe unavailable to the plant. The analytical methods used here could not distinguish active and total Fe pools.

Root B concentration was higher in rhododendrons grown in high pH nutrient solutions than in rhododendrons grown in low pH solutions. In agreement with previous research in blueberry [7], leaf B concentrations were lower in plants grown in high pH solutions than in plants grown in optimum pH solutions. This pattern suggests that plants are either accumulating B in the roots or translocating B from the shoots to roots. B retranslocation is species-dependent [32]. Thus, additional research will be necessary to determine if rhododendron can translocate B to the roots, leading to the observed pattern.

Unlike with other species [33,34,35], high pH nutrient solution led to a nuanced oxidative stress response in rhododendron. Oxidative status depends on the balance between reactive oxygen species (ROS) and antioxidants in plant cells. Previous research has shown that high pH can disrupt this balance by promoting ROS production [35] and limiting the availability of the micronutrient cofactors required by antioxidant enzymes [36,37]. Here, high pH nutrient solutions increased Fe concentration but decreased Cu and Zn concentrations in the roots. Fe excess can lead to ROS production through Fenton reactions [38]. Nevertheless, rhododendron grown in high pH nutrient solutions did not exhibit increased lipid peroxidation—a common assessment of oxidative stress. In agreement with this, enzymatic (catalase) and non-enzymatic (proline) antioxidants were generally not affected by nutrient solution pH. Considering the sparse sampling for oxidative status in this experiment, future research should expand on these observations.

In conclusion, high pH nutrient solutions cause widespread stress symptoms in rhododendron. Plants grown in high pH solutions exhibited diminished growth, abnormal root morphology, and impaired root function. Concentration, content, and uptake of most nutritional elements were affected by high pH solutions. S and Mn deficiencies likely limited plant growth. Altogether, these results indicate that high pH stress directly impacts roots and, through the resulting nutrient deficiencies, indirectly affects the rest of the plant.

## 4. Materials and Methods

### 4.1. Plant Material and Cultivation

One-year-old liners (average FW 36.70 ± 1.90 g) of “Mardi Gras” rhododendron (*Rhododendron yakushimanum* ssp. yakushimanum “Koichiro Wada” × *Rhododendron griersonianum* “Vanessa”) were acquired from a commercial nursery. Roots were washed clean of substrate using tap water. Then, plants were transplanted to 2.0-L bottles filled with a continuously-aerated nutrient solution as per Darnell and Cruz Huerta [39]. The nutrient solution contained 0.5 mM KNO_3_, 0.5 mM K_2_HPO_4_, 1.0 mM MgSO_4_, 0.5 mM CaCl_2_, 45 μM H_3_BO_3_, 45 μM Fe (delivered as Sequestrene 330™ (Becker Underwood, Inc.)), 10 μM MnSO_4_, 10 μM ZnSO_4_, 0.3 μM CuSO_4_, and 0.2 μM Na_2_MoO_4_. The nutrient solution was buffered using 5.0 mM 2-(N-morpholino) ethanesulfonic acid (MES). Nutrient solutions were changed on a weekly basis to maintain concentrations nearly constant. When necessary, deionized water was added to the bottles to compensate for water loss due to evapotranspiration.

Plants were acclimated to the hydroponic system for 22 days. During this period, nutrient solution was maintained at pH 5.5. Subsequently, plants were weighed and randomly assigned to one of two treatment groups. Half of the plants remained in nutrient solution at pH 5.5, while the other half were transferred to nutrient solutions at pH 6.5. Nutrient solution pH was adjusted using KOH or HCl as necessary. The treatment period lasted 49 days. A timeline detailing the timing of data collection can be found in Figure S2.

The experiment was conducted in a walk-in growth chamber equipped with LED lights (K5 series model XL750, Kind Lights, Santa Rosa, CA) delivering an average photosynthetic photon flux of 278 µmol m^−2^ s^−1^. Average temperature was 22.29 °C and average relative humidity was 70.98% during the experiment.

### 4.2. Enzymatic Measurements

Activity of the rate-limiting enzymes for NO_3_^−^, Fe, and P uptake was measured on root samples collected during the treatment period. These enzyme measurements have been used to characterize nutrient uptake responses in calcifuge plants [39,40,41].

Nitrate reductase activity was measured as per Darnell and Cruz-Huerta [39] with modifications. Approximately, 40 mg of root tips was collected in tubes containing 0.2 mM CaSO_4_ on ice. Two root samples per plant were placed in tubes containing an assay solution composed of 100 mM KH_2_PO_4_ at pH 7.5, 30 mM KNO_3_ and 2% v/v 1-propanol. Roots were removed from one sample per plant and used to assess root NO_2_^−^ concentration at the start of the assay. The second root sample from each plant was incubated under continuous shaking (540 rpm) for 60 min at 31 °C in the dark. Then, 150 μL of assay solution was mixed with 150 μL sulfanilamide (1% w/v in 1.5 N HCl) and 150 μL N-(1-naphthyl)-ethylenediamine dihydrochloride (0.02% w/v in 0.2 N HCl) and incubated again at 22 °C for 30 min in the dark. Finally, absorbance (540 nm) of the reacted solution was measured using a microplate reader (Synergy HT, BioTek Instruments, Winooski, VT, USA). NO_2_^−^ concentration was quantified using a standard curve prepared with KNO_2_. Nitrate reductase activity was measured 28 and 48 days after the start of the treatment period.

Ferric chelate reductase activity was measured as per Nunez et al. [42]. Root samples were collected as described above. Root samples were blotted dry and transferred to an assay solution containing 0.2 mM CaSO_4_, 5.0 mM MES at pH 5.5, 0.1 mM ethylenediaminetetraacetic acid iron (III) sodium salt, and 0.2 mM bathophenanthroline disulfonic acid disodium salt hydrate. Roots were incubated under continuous shaking (540 rpm) for 60 min at 22 °C in the dark. After incubation, absorbance (535 nm) of assay solution aliquots was measured using a microplate reader. Fe^2+^ concentration was computed using a molar extinction coefficient of 2.2 × 10^4^ M^−1^ cm^−1^ [43]. Assay solution samples incubated without roots and without Fe were used as controls. Ferric chelate reductase activity was measured 16 and 47 days after the start of the treatment period.

Acid phosphatase activity was measured as per Tabatai and Bremner [44]. Root tip samples were collected in tubes containing 50 mM sodium acetate at pH 5.5 on ice. Following, samples were blotted dry and transferred to an assay solution containing 50 mM sodium acetate at pH 5.5 and 270 mM p-nitrophenol. Roots were incubated under continuous shaking (540 rpm) for 30 min at 30 °C in the dark as per Zhang et al. [45]. After incubation, phosphatase activity was inhibited by adding 0.50 M NaOH and vortexing. Absorbance (405 nm) of inhibited solution was measured using a microplate reader. p-nitrophenol concentration was computed using a molar extinction coefficient of 1.83 × 10^4^ M^−1^ cm^−1^. Acid phosphatase activity was measured 22 and 44 days after the start of the treatment period.

### 4.3. Stress Response Measurements

Membrane integrity was assessed through the electrolyte leakage method as per McKay and White et al. [46]. Root samples were collected in aliquots of the nutrient solution in which they were growing. Then, roots were blotted and weighed before incubation in 16 mL deionized H_2_O. After 24 h of incubation under continuous shaking (130 rpm), assay solution electrical conductivity was measured with a standardized meter (model XL30, Fisher Scientific Inc., Waltham, MA). Then, roots and assay solution were autoclaved for 60 min at 121 °C and electrical conductivity was measured again. Control tubes containing no roots were incubated and measured identically. Relative electrolyte leakage was expressed as a percentage of total electrolytes extracted during the incubation period. Membrane integrity was measured 21 and 49 days after the start of the treatment period.

Root tip samples for lipid peroxidation, catalase activity, and proline concentration measurements were flash frozen using liquid N and stored at −80 °C until processing. Samples were collected twice during the experiment to minimize root injury. Approximately, 40 mg of frozen root tips were used to measure lipid peroxidation using the malondialdehyde method as per Shen et al. [47]. Root tissue was disrupted in tubes containing 1 mL of 5% trichloroacetic acid and two 3mm metal beads at a frequency of 1/30 Hz for 90 s (TissueLyser II, QIAgen GmbH, Hilden, Germany). Samples were centrifuged at 12,000× *g* for 20 min at 4 °C. Supernatant was collected and split into three 200 uL aliquots. Samples were reacted with 200 µL 0.67% thiobarbituric acid at 99 °C for 30 min. Tubes were placed on ice to halt the reaction, and then centrifuged at 10,000× *g* for 10 min at 4 °C. Absorbance (532 nm and 600 nm) of the supernatant was measured in a microplate reader. Control tubes containing no roots were incubated and measured identically. Malondialdehyde concentration was calculated using molar extinction coefficient 155 mM^−1^ cm^−1^. Lipid peroxidation was measured 19 and 33 days after the start of the treatment period.

Approximately 100 mg of frozen root tips was used to measure catalase activity. Roots were disrupted as detailed above in tubes containing 600 µL extraction solution containing 0.20 M potassium phosphate buffer (pH 7.0) and 0.1 mM ethylenediaminetetraacetic acid. Samples were then centrifuged at 15,000× *g* for 20 min at 4 °C. Supernatant was collected and transferred to a new tube. The process was repeated after adding 400 µL of extraction solution and centrifuging at 15,000× *g* for 15 min at 4 °C. Supernatant from both extractions was pooled and diluted to 75% with 50 mM potassium phosphate buffer at pH 7.0 (dilution solution). Catalase activity was measured as H_2_O_2_ detoxification using the pertitanic acid method as per Hadwan and Khabt [48]. In triplicate reactions, 25 µL of dilute supernatant was mixed with 250 µL of peroxide solution (30 mM hydrogen peroxide in dilution solution) and incubated for 3 min at 37 °C. Then, the reaction was stopped by adding 750 µL of development solution (0.1% TiCl_4_ in 20% H_2_SO_4_). Absorbance (405 nm) of the developed solution was measured in a microplate reader. Control tubes containing no supernatant and no peroxide were incubated and measured identically. H_2_TiO_4_ concentration was calculated using molar extinction coefficient 689 M^−1^ cm^−1^. Catalase activity was measured 4 and 19 days after the start of the treatment period. Five reactions exhibited negative values on each sampling date. Negative values were removed from the analysis, leading to n < 10 and uneven sample size between treatments.

Approximately 40 mg of frozen root tips were used to measure proline concentration using the ninhydrin method as per Bates et al. [49]. Acidic ninhydrin stock was prepared within 24 h of the assay using 1.25 g of ninhydrin stored under inert gas, 30 mL of glacial acetic acid, and 20 mL of 6 M orthophosphoric acid. Roots were disrupted, as detailed above, in tubes containing 3% sulfosalicylic acid. Disrupted samples were centrifuged at 16,000× *g* for 5 min at room temperature. Then, 100 μL of supernatant was mixed with 100 μL of 3% sulfosalicylic acid, 200 μL of acidic ninhydrin stock, and 200 μL of glacial acetic acid. Tubes were vortexed and incubated at 96 °C for 60 min. The reaction was terminated by placing tubes on ice. The chromogen was collected with 500 μL of toluene in a 5 min incubation at room temperature. Absorbance (520 nm) of the toluene fraction was measured in a microplate reader. Proline concentration was calculated using a standard curve. Proline concentration was measured 19 and 33 days after the start of the treatment period.

### 4.4. Nutrient Uptake

Nutrient uptake was measured as element depletion from nutrient solutions as per Nunez et al. [31]. Plants received 2 L of nutrient solution at the start of each week. Nutrient solution volume was measured after 7 days of hydroponic cultivation. Nutrient solution samples were collected at the start and end of the week. Samples were acidified with 1 drop of 12 M HCl and submitted to an institutional laboratory (University of Florida Institute of Food and Agricultural Sciences Analytical Research Laboratory, Gainesville, FL) for elemental analysis by inductively coupled plasma–atomic emission spectrometry [50]. Nutrient content at the start and end of the week were computed by multiplying nutrient solution volume and concentration. Differences in nutrient content between the start and end of the week were assumed to reflect plant nutrient uptake.

### 4.5. Destructive Harvest

Plants were destructively harvested at the end of the treatment period. Whole plant and organ by organ fresh weight (FW) were measured after blotting root systems dry. Then, relative growth rate was calculated as the difference between the natural logarithm of the fresh weight at the start and the end of the treatment period [51]. Leaf chlorosis was assessed using two different greenness meters: one based on light transmittance (SPAD 502, Konika Minolta, Osaka, Japan) and one based on image analysis (FieldScout Green Index+, Spectrum Technologies Inc., Aurora, IL, USA). Leaves were laid flat on a white background and photographed using a mobile phone camera (iPhone X, Apple Inc., Cupertino, CA, USA) placed 48 cm away. Then, total leaf area was measured using ImageJ version 1.51 [52]. Roots were observed and photographed with a digital microscope (Q-Scope 13200 Lite, Euromex Microscopen B.V., Arnhem, The Netherlands). Following, the tissue was dried to a constant weight at 72 °C, ground until it passed through a size-20 mesh and submitted for elemental analysis at a commercial laboratory (Waters Laboratory, Camila, GA, USA).

### 4.6. Data Analysis

The experiment followed a completely randomized design with one treatment (nutrient solution pH) and two levels (pH 5.5 and pH 6.5). There were 10 single-plant replications per treatment. Data were analyzed through one-way ANOVA using package agricolae [53] in R (version 3.6.2; R Foundation for Statistical Computing, Vienna, Austria). Where the sample size was uneven, treatments were compared though one-way ANOVA using the type III sum of squares with the car package [54]. Data were illustrated using package ggplot2 [55].

## Figures and Tables

**Table 1:** Biomass accumulation of “Mardi Gras” rhododendron grown in nutrient solutions with optimum (pH 5.5) or high pH (pH 6.5) for 49 days. Data reported are means ± standard errors.

Nutrient Solution pH	Root Dry Weight (g)	Stem Dry Weight (g)	Leaf Dry Weight (g)	Total Dry Weight (g)	Root to Shoot Ratio	Total Leaf Area (cm^2^)
5.5	10.50 ± 0.64	4.19 ± 0.46	4.88 ± 0.35	20.38 ± 1.35	1.15 ± 0.04	557.77 ± 33.56
6.5	8.05 ± 0.54	4.07 ± 0.45	3.35 ± 1.69	15.93 ± 1.51	1.09 ± 0.09	412.31 ± 18.75
*p*-value ^1^	0.009	0.857	0.042	0.049	0.649	<0.001

^1^ Data were analyzed through one-way analysis of variance.

**Table 2:** Nutritional element concentration in roots and leaves of “Mardi Gras” rhododendron grown hydroponically in optimum (pH 5.5) and high pH (pH 6.5) nutrient solutions.

Organ	Nutrient Solution pH	Macronutrient Concentration (%)					Micronutrient Concentration (ppm)				
		N	P	Ca	Mg	S	Fe	Cu	Mn	Zn	B
Roots	5.5	1.29	0.31	0.48	0.22	0.22	3439.90	57.40	881.80	162.70	16.50
	6.5	0.98	0.35	0.56	0.24	0.18	3953.30	16.36	742.82	29.91	23.76
	*p-*value ^1^	0.022	0.627	0.262	0.405	0.002	0.026	<0.001	0.315	<0.001	<0.001
Leaves	5.5	0.83	0.18	0.47	0.24	0.28	47.80	3.10	179.60	24.30	31.60
	6.5	0.94	0.14	0.45	0.20	0.23	50.09	3.45	103.00	24.00	26.09
	*p-*value ^1^	0.002	0.002	0.196	0.025	0.002	0.687	0.304	<0.001	0.896	0.002

^1^ Data were analyzed through one-way analysis of variance.

**Table 3:** Nutritional element content in roots and leaves of “Mardi Gras” rhododendron grown hydroponically in optimum (pH 5.5) and high pH (pH 6.5) nutrient solutions.

Organ	Nutrient Solution pH	Macronutrient Content (g)					Micronutrient Content (mg)				
		N	P	Ca	Mg	S	Fe	Cu	Mn	Zn	B
Roots	5.5	0.137	0.033	0.052	0.024	0.024	3.611	0.060	0.910	0.169	0.017
	6.5	0.079	0.027	0.046	0.018	0.014	3.364	0.014	0.601	0.025	0.018
	*p-*value ^1^	<0.001	0.149	0.470	0.077	<0.001	0.596	<0.001	0.007	<0.001	0.688
Leaves	5.5	0.0439	0.009	0.022	0.010	0.012	0.022	0.001	0.075	0.011	0.014
	6.5	0.041	0.006	0.020	0.007	0.008	0.016	0.001	0.036	0.008	0.009
	*p-*value ^1^	0.839	0.16	0.808	0.035	0.007	0.084	0.480	0.005	0.185	0.065

^1^ Data were analyzed through one-way analysis of variance.

## Supplementary Materials

The following are available online at https://www.mdpi.com/2223-7747/9/8/1019/s1, Figure S1: Root stress measurements in ‘Mardi Gras’ rhododendron, Figure S2: Timeline of data collection, Table S1: Activity rates of enzymes involved in nutrient uptake.
