# Supplementary material for: High pH Stress Affects Root Morphology and Nutritional Status of Hydroponically Grown Rhododendron (Rhododendron spp.)
Source: Plants (Basel). 2020 Aug 12;9(8):1019. doi: 10.3390/plants9081019 (PMC7465443; doi:10.3390/plants9081019)
Supplement: Supplementary file 1 [file plants-09-01019-s001.pdf]

Table S1. Activity rates of enzymes involved in nutrient uptake in the roots of 'Mardi Gras' rhododendron grown hydroponically at optimal (pH 5.5) and high (pH 6.5) nutrient solution pH.

Nutrient solution pH	Nitrate reductase (nmol NO_2^- g^{-1} h^{-1})		Ferric chelate reductase (μmol Fe^{2+} g^{-1} h^{-1})		Acid phosphatase (μmol p-nitrophenol g^{-1} h^{-1})	
	**28 DAT [1]**	**48 DAT**	**16 DAT**	**47 DAT**	**22 DAT**	**48 DAT**
5.5	46.74	231.60	7.187	0.99	1.64	2.94
6.5	30.89	57.06	5.354	0.80	1.60	2.13
p-value [2]	0.087	0.015	0.136	0.194	0.663	0.049

[1] DAT = days after the start of treatment. [2] Data were analyzed through one-way analysis of variance.

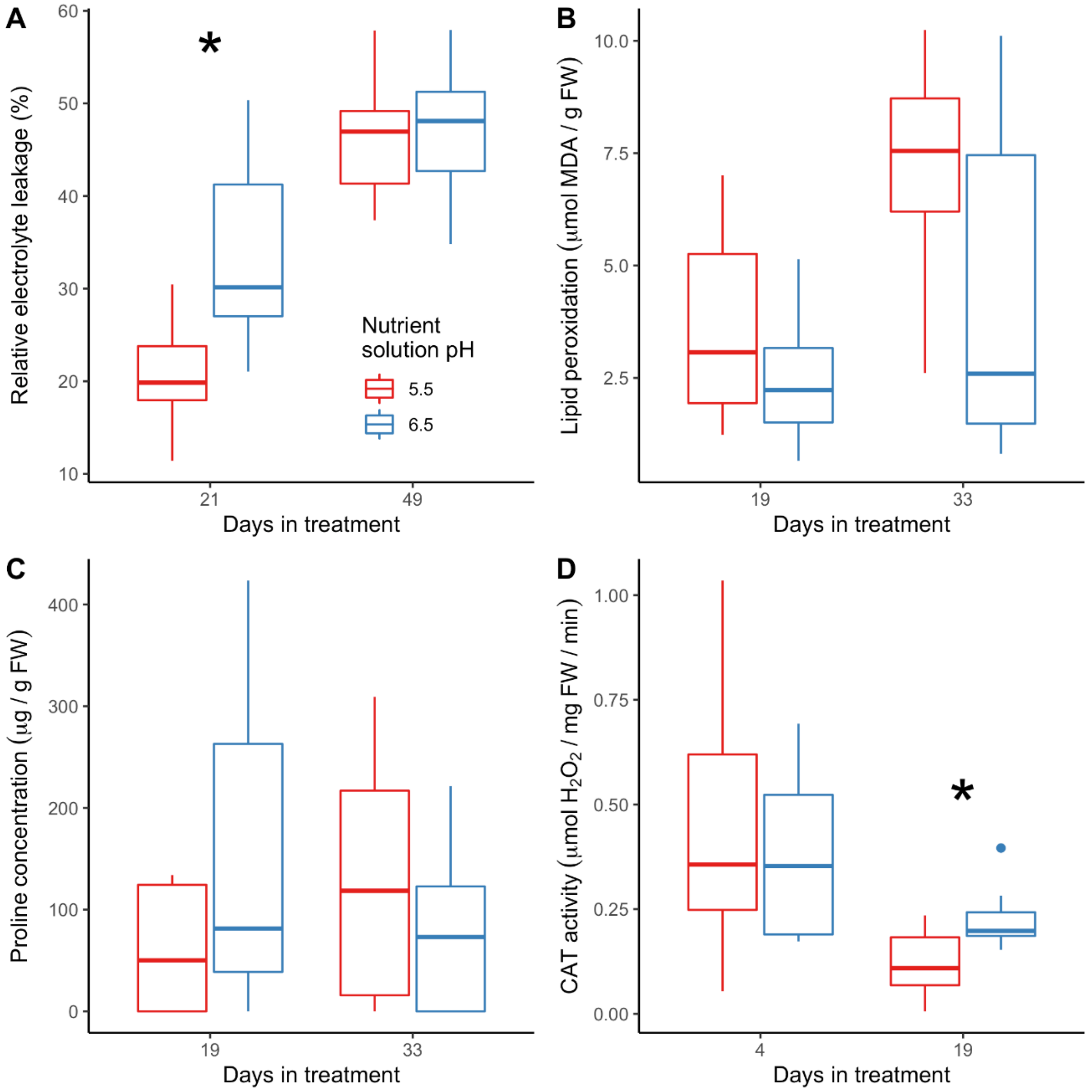

Figure S1. Root stress measurements in 'Mardi Gras' rhododendron grown in nutrient solution at. pH 5.5 or pH 6.5. Electrolyte leakage (**A**) and catalase (CAT) activity (**D**) increased three weeks after transfer to high pH nutrient solution. Lipid peroxidation (**B**) and proline concentration (**C**) were not affected by nutrient solution pH. Asterisks indicate significant differences at $\alpha = 0.05$, as determined by ANOVA.

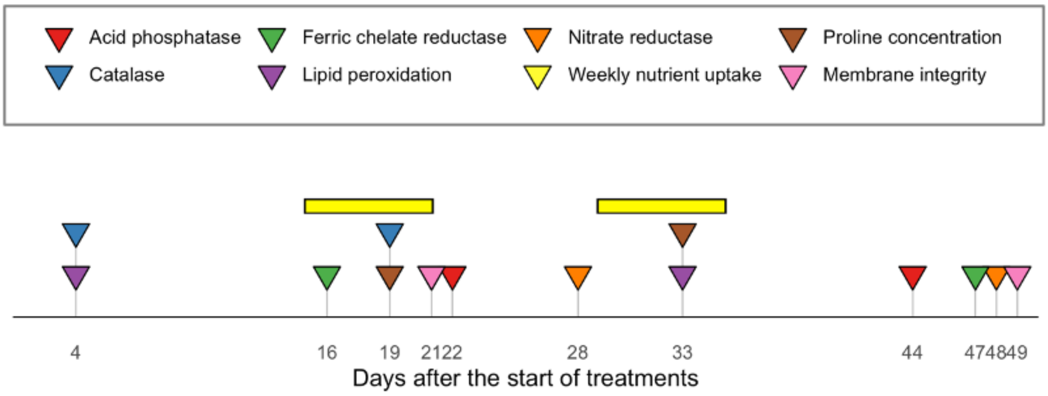

Figure S2. Data collection timeline in a hydroponic experiment where 'Mardi Gras' rhododendron was grown in nutrient solution at pH 5.5 or pH 6.5 for 49 days.
